# Entrainment to Periodic Initiation and Transition Rates in a Computational Model for Gene Translation

**DOI:** 10.1371/journal.pone.0096039

**Published:** 2014-05-06

**Authors:** Michael Margaliot, Eduardo D. Sontag, Tamir Tuller

**Affiliations:** 1 School of Electrical Engineering and the Sagol School of Neuroscience, Tel-Aviv University, Tel-Aviv, Israel; 2 Dept. of Mathematics and Cancer Institute of New Jersey, Rutgers University, Piscataway, New Jersey, United States of America; 3 Dept. of Biomedical Engineering and the Sagol School of Neuroscience, Tel-Aviv University, Tel-Aviv, Israel; Universitat Pompeu Fabra, Spain

## Abstract

Periodic oscillations play an important role in many biomedical systems. Proper functioning of biological systems that respond to periodic signals requires the ability to synchronize with the periodic excitation. For example, the sleep/wake cycle is a manifestation of an internal timing system that synchronizes to the solar day. In the terminology of systems theory, the biological system must entrain or phase-lock to the periodic excitation. Entrainment is also important in synthetic biology. For example, connecting several artificial biological systems that entrain to a common clock may lead to a well-functioning modular system. The cell-cycle is a periodic program that regulates DNA synthesis and cell division. Recent biological studies suggest that cell-cycle related genes entrain to this periodic program at the gene translation level, leading to periodically-varying protein levels of these genes. The ribosome flow model (RFM) is a deterministic model obtained via a mean-field approximation of a stochastic model from statistical physics that has been used to model numerous processes including ribosome flow along the mRNA. Here we analyze the RFM under the assumption that the initiation and/or transition rates vary periodically with a common period 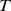. We show that the ribosome distribution profile in the RFM entrains to this periodic excitation. In particular, the protein synthesis pattern converges to a unique periodic solution with period 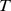. To the best of our knowledge, this is the first proof of entrainment in a mathematical model for translation that encapsulates aspects such as initiation and termination rates, ribosomal movement and interactions, and non-homogeneous elongation speeds along the mRNA. Our results support the conjecture that periodic oscillations in tRNA levels and other factors related to the translation process can induce periodic oscillations in protein levels, and may suggest a new approach for re-engineering genetic systems to obtain a desired, periodic, protein synthesis rate.

## Introduction

External and internal periodic oscillations play an important role in intracellular and extracellular biomedical systems and have attracted enormous research interest (see e.g. [1] and the references therein). Proper functioning of cells that are exposed to such periodic signals requires internal biological mechanisms that are able to *synchronize* with the periodic excitation. In the terminology of systems theory, the biological system must *entrain* or phase-lock to the periodic excitation. In other words, in response to a periodic excitation with period 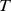 the system's internal state converges to a periodic signal with period 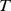.

Entrainment in biological systems (sometimes called phase locking [2]) and, more generally, biological oscillators and rhythms have recently attracted enormous attention (see *e.g.* [3]–[5] and the references therein). For example, the sleep/wake cycle is a manifestation of an internal timing system that entrains to the 24 hours period of the solar day using a visual pathway connecting the retina to the suprachiasmatic nucleus (SCN) [6].

Entrainment is also important in *synthetic biology*. For example, most hormones in the body are released in periodic pulses. Glucocorticoid secretion, for instance, has a circadian and ultradian pattern of release. Synthetic biological oscillators may be used to mimic these periodic release patterns in the administration of synthetic hormones to patients suffering from glucocorticoid-responsive diseases, thus improving therapeutic effectiveness [7].

The design of robust synthetic biological oscillators is also the first step for applications such as clocks that synchronize in order to coordinate intracellular behavior, and artificial platforms that can measure the genomic response to an oscillatory excitation [8].

### Entrainment at the intra-cellular gene expression level

Proteins are “tiny machines” performing a vast array of functions within living organisms including intra- and inter-cellular signaling transduction, immunological response against pathogens, movement of cells and tissues, facilitation of biochemical reactions, structure and support of the cell and tissue, and transport. Regions in the DNA, called genes, encode the information needed to produce proteins. *Gene expression* is the process by which the information inscribed in the genes is converted into proteins. The major steps of gene expression are transcription, translation, and mRNA and protein turnover [9].

The cell-cycle is a periodic program that regulates DNA synthesis and cell division. Proper execution of the cell-cycle requires the expression and activation of key proteins at specific times along the period. This process must be tightly regulated, as perturbations in cell-cycle progression can lead to apoptosis or cancer.

Recently, there is growing evidence that protein levels of cell-cycle related genes can be regulated not only via transcription (see, for example, [10]) but also via the translation elongation step. Higareda-Mendoza and Pardo-Galvan [11] investigated the role of human translation initiation factor 3 (eIF3) in cell-cycle control of A549 cells. They reported that eIF3f expression oscillates during cell-cycle, with one maximum expression peak in the early S phase and a second during mitosis. Their interpretation is that eIF3f is a translational modulator that selects mRNAs at specific cell-cycle phase time points.

Frenkel-Morgenstern et al. [12] have shown that cell-cycle regulated genes tend to include non-optimal codons, *i.e.*, codons that are rare and are recognized by tRNA molecules with low intra-cellular abundance, and thus with a low translation rate. These codons create “bottlenecks” in the translation process and thus their slow translation rate becomes rate limiting. They argue that periodicity in the tRNA levels of these codons induces periodicity in the translation rate of these genes. The fact that cell-cycle regulated genes display different codon preferences at different phases of the cell-cycle supports the conjecture that cells exploit non-optimal codons to generate cell-cycle-dependent dynamics of proteins via the translation process. In other words, the translation process *entrains* to the excitation generated by periodically varying tRNA levels.

In another recent study, Patil et al. [13] have reported an additional mechanisms by which cell-cycle can be regulated via translation. The ribonucleotide reductase (RNR) complex plays an important role in regulating cell-cycle transitions and in DNA damage response. They showed that the levels of 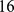 tRNA modifications, and thus the translation efficiencies of different codons *oscillate during cell-cycle*; in addition, these oscillations match the wobble interaction needed for translating codon of genes such as RNR1. Their results imply that translation regulation has a direct role in cell-cycle related oscillations.

Two other recent studies [14], [15] suggest that non-optimal codon usage during translation affect the expression, structure, and functioning of proteins, and are particulary important in the context of circadian clocks.

Periodicity in gene expression that is related to periodic processes, such as the cell-cycle and biological clocks, is regulated at all the different gene expression stages. This includes transcription, translation, and post-translational regulation. The related regulation mechanisms include dozens (or even hundreds) of genes and proteins that interact with each other in ways that we are only beginning to unveil. Usually these networks of interactions include a few redundant mechanisms of oscillation regulation [14]–[20]. For example, it was suggested that cell-cycle regulation includes negative feedback oscillators. These can include for example the interconnection of two genes where the first gene up regulates the second, and the second down regulates the first [16]. Another possible regulation mechanism is via control of the transcription rate of tRNA genes (and other genes), resulting in oscillations in intra-cellular tRNA levels [12]. Since the decoding time of codons is affected by the available levels of the tRNA molecules recognizing them (see, for example, [21], [22]), this may eventually lead to oscillations in the decoding times of different codons.

It may seem natural to assume that periodic variations, with period 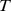, in the initiation rate and/or the decoding times of different codons will lead to a periodic protein production rate with the same period 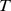. However, this assumption is actually quite strong. Indeed, these factors affect the protein synthesis rate via the dynamics of the translation mechanism, and not every dynamical system entrains to periodic excitations. Here we analyze this problem using a computational model for translation-elongation.

### Entrainment in a computational model for translation

High-throughput experiments provide more and more data on the translation process. Computational models of translation are needed to organize, understand, and connect this data to various biophysical aspects of translation [23]–[28]. Understanding the *dynamics* of gene expression, and not only the static information encoded in the genes, is vital in order to understand how the biological components work together to comprise functioning cells and organisms. Developing a deeper understating of the dynamics of translation may thus have implications in many fields of science including human health [29]–[35], biotechnology [36]–[40], evolution [31], [41]–[47], functional genomics [48]–[54], and systems biology [33], [51], [55]–[59]. Recent reviews related to translation may be found in [38], [42], [60].

A rigorous analysis of these models can deepen understanding of the translation process, assist in integrating the vast amount of empirical findings, lead to efficient algorithms for optimizing gene translation for various biotechnological goals, and help to improve the fidelity and predictive ability of the models. In the near future, this will enable building syntectic biological devices that are based on re-engineering biological mechanisms and specifically gene expression.

A newly developed technique, called *ribosome profiling* [61], [62], provides indications on the occupancy of codons by ribosomes along the mRNA molecules in vivo. This breakthrough has led to a renewed interest in computational models for translation (see e.g., [63]–[65]).

Reuveni *et al.* [66] considered a deterministic model for translation called the *ribosome flow model* (RFM). This model is a deterministic approximation of an important model from statistical physics, called the *asymmetric simple exclusion process* (ASEP), that is the standard mathematical model for ribosome flow. ASEP has also been used to model and analyze many other systems and processes, including traffic flow, molecular motors, surface growth, the movement of ants, and more [67].

In this paper, we study the dynamical behavior of the RFM under the assumption that some or all of its parameters vary periodically, with a common period 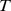. This models periodically time-varying initiation and/or transition rates along the mRNA. We refer to this model as the *periodic ribosome flow model* (PRFM).

#### Main results and their implications

Our main result shows that the PRFM entrains to a periodic excitation. In other words, the PRFM admits a unique periodic solution with period 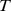, and all the state-variables converge to this solution. This means that all the ribosome densities converge to a periodic pattern with period 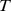 and, in particular, the protein synthesis rate converges to a periodic pattern. To the best of our knowledge this is the first proof of entrainment in a non-trivial mathematical model for translation.

Our results suggest that entrainment takes place in particular in the case where the codon decoding rates (called transition rates in the RFM) are constant, and the initiation rate is 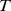-periodic. Similarity, entrainment takes place if the initiation rate is constant and *some* of the transition rates are 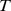-periodic. From a biophysical perspective, this suggests that periodic oscillations of the translation rate (and thus protein abundance) can be induced in various ways including: 1) oscillations of factors related to the initiation step such as the mRNA levels of genes, the abundance of ribosomes, and the abundance of initiation factors; and 2) oscillations of factors related to the elongation step such as the abundance of elongation factors and tRNA genes. Specifically, oscillations in the abundance of a *single* tRNA gene is enough to induce oscillations in the translation rate and protein abundance.

These results have several implications. First, they support the conjecture that cell-cycle dependent dynamics of proteins may be obtained by entrainment in the translation process. Moreover, the biological mechanism can generate a periodic production rate relatively easy; it is enough to vary just one tRNA abundance in a periodic manner. However, in the PRFM entrainment takes place whenever the initiation/transition rates vary periodically (with a common period) *regardless* of their amplitude. This suggests that the bottleneck argument in [12] is not necessarily needed.

Second, in the context of synthetic biology our results may lead to new mechanisms for generating various syntectic devices at the *translation* level. Several recent studies considered the design of synthetic biological oscillators, mostly based on manipulating aspects related to transcription (see, e.g. [1], [8], [68]–[71]). The authors of [13] raise the question of why would cells regulate translation using codon usage and changes in tRNA modification status. They hypothesize that a rapid change in the abundance of tRNA modifications may allow cells to *quickly* reset the translation speed of existing transcripts, and thus respond quickly to stress or other changes in environmental conditions. If this is indeed so then developing synthetic biology devices based on entrainment at the translation level may have unique advantages.

#### Mathematical tools

In order to make this paper more accessible, we now briefly explain the main mathematical tools that are used in analysis.

Proving entrainment in non-linear dynamical systems is non-trivial. One standard approach is based on *contraction theory* [3], [72], [73]. A dynamical system is called contracting if the distance between trajectories emanating from any two initial conditions quickly decreases with time (more precisely, it decreases at an exponential rate). This means that the information about the initial condition is “quickly forgotten”.

Consider a system that is periodically excited with a period 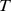. Assuming that the trajectories remain bounded, it is possible to show that the system admits a periodic solution with period 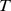. Consider two trajectories, one emanating from an initial condition on this periodic solution, and the second from some arbitrary initial condition. If the system is also contracting then these trajectories must converge to one another, so all trajectories converge to the periodic solution. This proves entrainment.

The proof of entrainment in the PRFM is based on these ideas. However, some additional analysis is needed, as the RFM is on the “verge of contraction”, yet it is not contracting on its entire state space.

The remainder of this paper is organized as follows. The next section briefly reviews the ASEP and RFM. The main results about entrainment in the PRFM are described in the Results section. The proofs are detailed in the Methods section. The Discussion section provides a summary, and describes possible directions for further research.

## Preliminaries: From ASEP to the RFM

An important computational model for translation elongation is the *Asymmetric Simple Exclusion Process* (ASEP) [25], [26], [74]. In this stochastic model particles hop, according to some probability function, between consecutive sites on a 1D lattice. Each site can be either occupied by a particle or not. Hops may take place only to a target site that is not already occupied by another particle (hence the term *simple exclusion*). The term *asymmetric* implies that there is some preferred direction of movement along the lattice. If motion is allowed only in one direction then ASEP is sometimes called the *totally asymmetric simple exclusion process* (TASEP). ASEP (and its many variants) is regarded as a paradigmatic model for non-equilibrium statistical mechanics and has been used to model and analyze various biological systems and processes, including intracellular transport, molecular motors, pedestrian dynamics and of course gene expression [75]–[80].

In TASEP models for translation each site in the lattice corresponds to a codon, the hopping particles are ribosomes, and their footprint includes 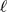 sites (in the case of translation, the footprint of a ribosome is usually 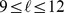 codons corresponding to 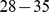 nt [61]). For example, a new particle (ribosome) can enter the lattice only if all the first 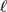 sites are all empty. Initiation time as well as the time a ribosome spends translating each codon are random variables (*e.g.*, with an exponential distribution), and are codon–dependent. Analysis of TASEP is based on determining the probability of steady-state configurations using matrix products (see the excellent survey paper [81]).

Reuveni *et al.* [66] recently considered a *deterministic* model for translation called the *Ribosome Flow Model* (RFM). The RFM is a finite-dimensional mean-field approximation of TASEP (see, e.g., [81], p. R345 and [82], p. 1919). The RFM includes 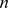 state-variables connected by a set of 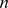 non-linear first-order differential equations:
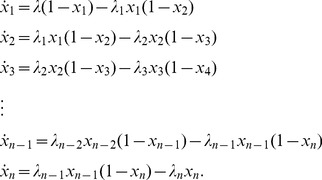
(1)The positive parameters 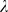 [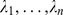] are called the initiation rate [transition rates]. The state-variable 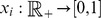 describes the “level of occupancy” of ribosomes at site 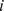 at time 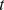, where 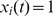 [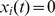] corresponds to the site being completely full [empty].

To explain this model, consider for example the equation for 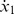, *i.e.* the change of “level of occupancy” at site 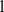. The term 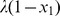 models the fact that ribosomes reach the first site with initiation rate 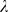, but their effective binding rate depends on how occupied site 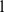 is. In particular, if 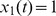, *i.e.* the site is completely full, the effective binding rate is zero. The term 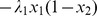 describes the rate of transition of ribosomes from site 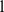 to the consecutive site 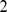.

Note that in ASEP each site may include either zero or one particles. In the RFM, the 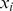
s correspond to averaged occupancy levels and therefore 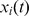 takes values in the closed interval 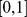.

We refer to
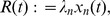
that is, the exit rate of ribosomes from the last site, as the *translation rate* at time 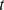.

Since the state-variables correspond to normalized occupancy levels, the initial condition 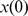 is always in the closed unit cube:
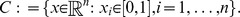
The simulation results in [66] show that TASEP and its mean-field approximation (the RFM) yield similar predictions of translation rates. For example, the correlation between their predictions over the set of endogenous genes of *S. cerevisiae* is 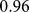. Important features of translation elongation that are captured in TASEP, for example, the sequential order of the codons, translation efficiency, the interaction between ribosomes and their jamming, the initiation and elongation rates, are also encapsulated in the RFM.

We now briefly summarize some known results on the dynamical behavior of the RFM. Let 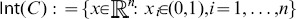 denote the interior of 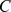, and let 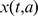 denote the solution of the RFM at time 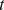 for the initial condition 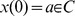. It has been noted in [83] that the RFM is a (tridiagonal) *monotone dynamical system* [84]. Combining this with the fact that 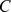 is an invariant set of the dynamics and a theorem of Smillie [85] yields the following result.

**Theorem 1** [*83*] *The RFM admits a unique equilibrium point* 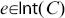, *and* 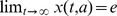 *for all* 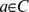.

This means that there exists a unique steady-state profile of ribosome distributions (and thus a unique translation rate). The trajectory starting from any initial distribution will converge to this steady-state profile. Changing the values of the positive parameters 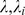 will not change this qualitative picture, only the steady-state profile.

Let 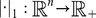 denote that 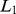 vector norm, *i.e.* 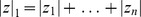. It has also been shown in [83] that the RFM is *non-expanding* with respect to the 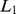 norm, that is,
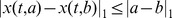
(2)for all 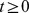 and all 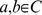. This means that the 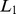 distance between two ribosome distribution profiles can never increase. It is worth noting that both Theorem 1 and (2) follow immediately from the more general results in this paper.

In some cases the transition rate along genes is constant [86], so the translation efficiencies of all the codons are identical. This happens, for example, when the rate limiting factor is the concentration of elongation factors and not the local features of the coding sequence, such as tRNA molecules or when there is a balance between the codon frequencies and tRNA levels [87]. In the context of the RFM, this can be modeled by considering the special case where
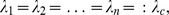
that is, the transition rates 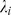 are all equal, and 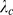 denotes their common value. Since this *Homogeneous Ribosome Flow Model* (HRFM) includes only two parameters, 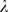 and 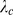, the analysis is considerably simplified. Ref. [88] analyzes the qualitative and quantitative dependence of 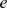 on the parameters 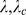 in the HRFM. Ref. [89] studied the HRFM when 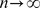, i.e. when the length of the mRNA chain goes to infinity. In this case, it is possible to obtain closed-form expressions for the equilibrium point using the theory of infinite 1-periodic continued fractions.

In eukaryotes the translation rate can affect the initiation rate via recycling of ribosomes. To model this, Ref. [90] has considered the RFM as a *control system*. Here 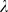 is replaced by a function 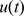 (the input), and an output 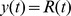 is added. It has been shown that this is a *monotone control system*, as defined in [91]. Also, analysis of the closed-loop system, obtained by closing the loop from 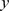 to 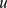 with positive linear feedback, has shown that several nice properties of the RFM hold also for the closed-loop system. In particular, there exists a unique globally asymptotically stable equilibrium point 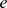. For the special case of equal 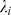
s, closed-form expressions relating the closed-loop system parameters and 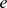 have been obtained.

## Results

**Figure 1:**
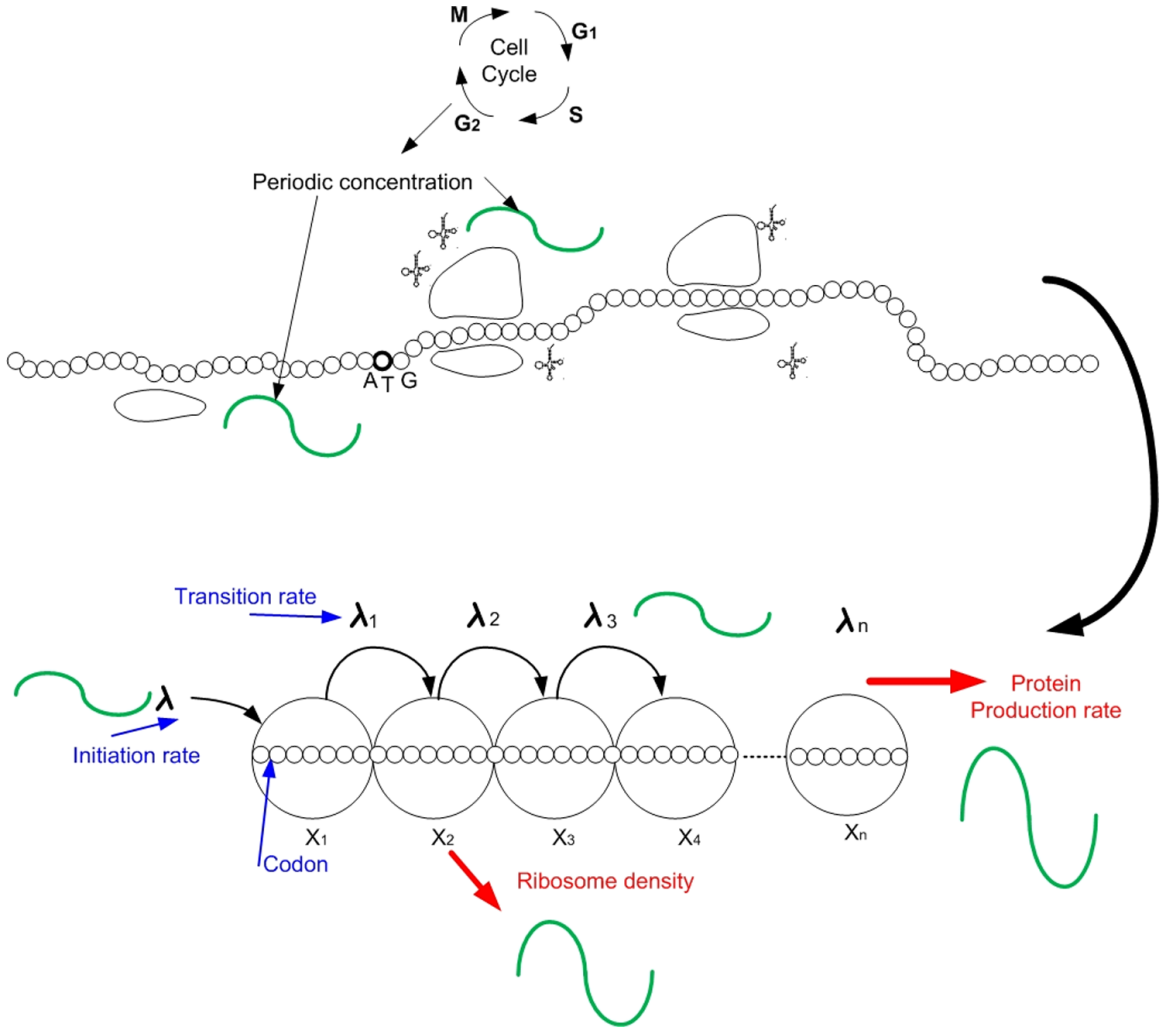
Upper part: the elongation rates of codons and the initiation rate are 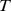-periodic, for example, due to signals related to the cell-cycle. Lower part: in the RFM, this is modeled by 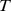-periodic rates 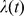 and 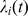 yielding the PRFM. Our main result shows that consequently the translation rate and ribosomal densities (the 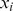
s) converge to a unique 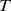-periodic solution.

A function 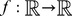 is called 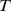-periodic if
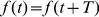
(3)for all 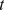. For example, 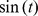 is 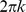 periodic, for all integers 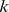. In this paper, we consider the behavior of the RFM (1) under the following assumptions (see Figure 1):

1. The initiation rate 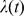 and transition rates 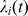, 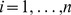, are continuous, strictly positive and uniformly bounded functions of time, *i.e.*, there exist 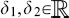 such that
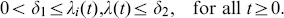
(4)
2. There exists a minimal 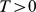 such that all these functions are 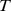-periodic.

We refer to this case as the *periodic ribosome flow model* (PRFM).

**Remark 1** Note that the PRFM includes in particular the case where some of these rates are constant, as a constant function is 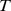-periodic for every 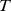. However, item 2) above implies that the case where all the rates are constant is ruled out, as then the minimal 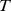 is zero. Indeed, this case is just the RFM.

The next example illustrates the dynamical behavior of the PRFM.

**Figure 2:**
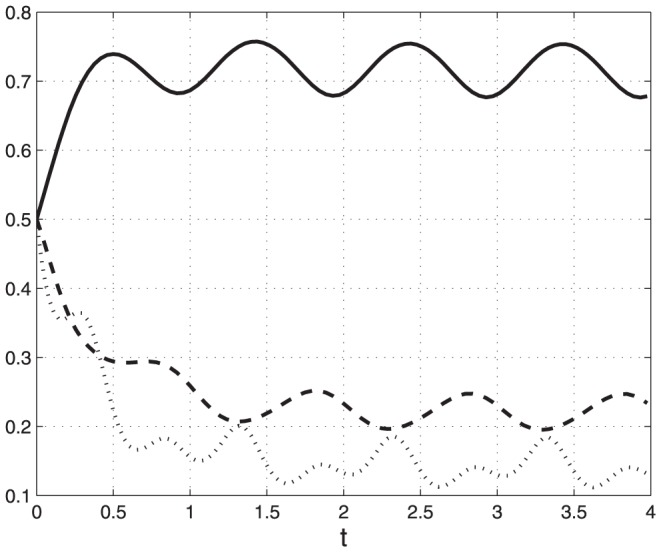
State-variables 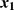
(*t*) [solid line]; 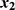
(*t*) [dashed]; and 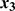
(*t*) [dotted] (y-axis) as a function of time (x-axis) in Example 1. All state-variable converge to a periodic signal with period 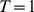.

**Example 1** Consider the PRFM with 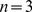,
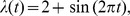


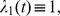


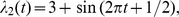


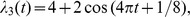



and initial condition 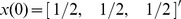. Note that all the rates here are periodic, with minimal common period 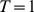. Figure 2 depicts 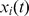, 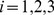, as a function of 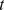. It may be seen that each state-variable converges to a periodic signal with period 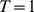.

The next example considers the case where only the initiation rate oscillates, and the constant transition rates are the rate-limiting factors.

**Figure 3:**
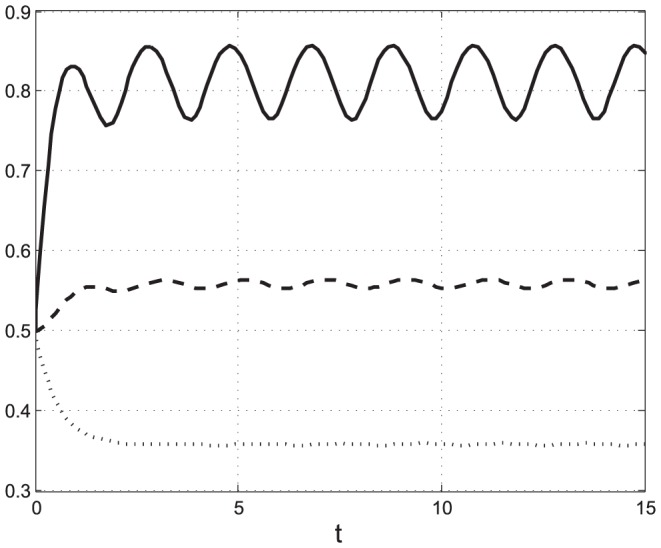
State-variables 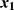
(*t*) [solid line]; 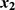
(*t*) [dashed]; and 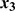
(*t*) [dotted] (y-axis) as a function of time (x-axis) in Example 2. The initiation rate is periodic with period 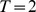, while the transition rates are constant and relatively small. All state-variable converge to a periodic signal with period 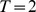, but the amplitude of the oscillations is considerably attenuated as it passes through the mRNA chain.

**Example 2** Consider the PRFM with 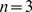,
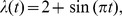


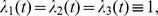
and initial condition 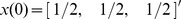. Note that here the transition rates are constant and relatively small, whereas the initiation rate is larger and periodic with period 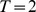. Figure 3 depicts 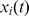, 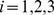, as a function of 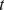. It may be seen that all the 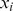
s converge to a periodic signal with period 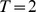. Note that the rate-limiting transition rates considerably attenuate the oscillations amplitude as they propagate through the mRNA chain. Here the small transition rates form the “bottleneck” in the process. This example demonstrates that entrainment takes place even when the rate-limiting factor is constant, as long as there exists at least one other factor that is periodic with 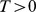. 

Our main result shows that the state-variables in the PRFM always entrain to a unique periodic solution. Let 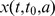 denote the solution of the PRFM at time 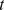 for the initial condition 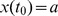.

**Theorem 2** *The PRFM admits a unique periodic solution* 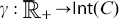, *with period* 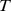, *and*

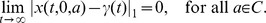



In other words, every trajectory converges to the unique periodic trajectory 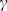. In particular, the translation rate 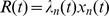 converges to the 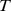-periodic function 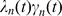.

By Remark 1, Theorem 2 holds in particular in the case where the transition rates 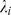, 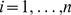, are constant, and the initiation rate is 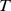-periodic (but not constant). Similarity, Theorem 2 also holds if the initiation rate is constant and *some* of the transition rates, 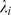, are 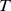-periodic (but not constant).

The stochastic nature of reaction events induces random noise in biochemical networks (see, for example, [92], [93]). As noted in [94], this becomes particularly important when there are few molecules in the system, as is often the case in a cell. It is natural to consider whether entrainment in the PRFM takes place also in the presence of noise. Our simulations suggest that this is indeed the case.

**Figure 4:**
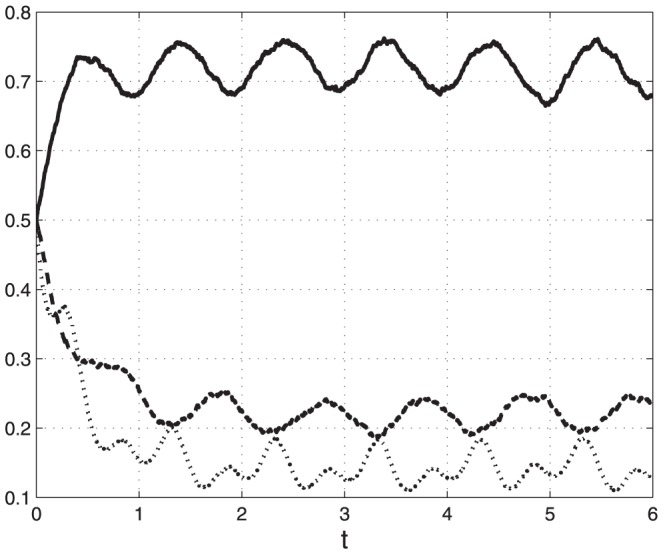
State-variables 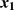
(*t*) [solid line]; 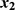
(*t*) [dashed]; and 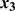
(*t*) [dotted] (y-axis) as a function of time (x-axis) in Example 3. The initiation and transition rates are periodic with a common period 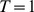, but with added random noise. It may be seen that each state-variable converges to a periodic signal with period 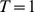, but with added noise.

**Example 3** Consider the PRFM with 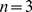,
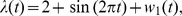


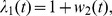


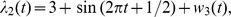


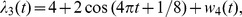
and initial condition 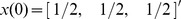. The 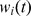
's are drawn as independent random values from the uniform distribution on the interval 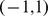 for all 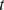. Note that this implies that all the rates remain positive for all 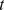. Figure 4 depicts 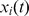, 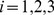, as a function of 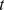. It may be seen that each state-variable still converges to a periodic signal with period 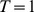, but with perturbations induced by the noise. 

Further study of entrainment in the PRFM in the presence of random perturbations is beyond the scope of this paper. However, we note that there exist theoretical results on contraction in the presence of random noise; see e.g. [95], [96].

As mentioned above, the RFM is a mean-field approximation of TASEP. Thus, our results suggest a natural question, namely, does TASEP entrain?

**Example 4** Consider a TASEP model with 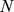 sites. When a particle in site 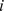 cannot hop because site 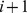 contains a particle, the next hopping time is determined by
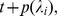
where 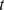 is the current time, and 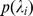 is a random variable drawn from the exponential distribution with mean parameter 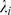. Recall that the exponential probability distribution function with mean 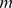 is given by
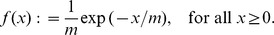
At the next hopping time, this particle hops unless the next site is full again, in which case a new hopping time is drawn.

We ran a simulation of this process with 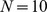, and final time 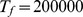. In the simulation, performed using MATLAB, time was discretized using a time step of 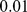. (In particular, the next hopping times are always rounded to a value 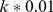, where 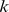 is an integer.) Initially, all sites are empty. The rates are
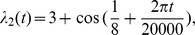


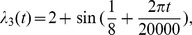
and 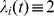 for all 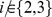. Note that all these rates are periodic with a common minimal period
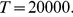
(5)


**Figure 5:**
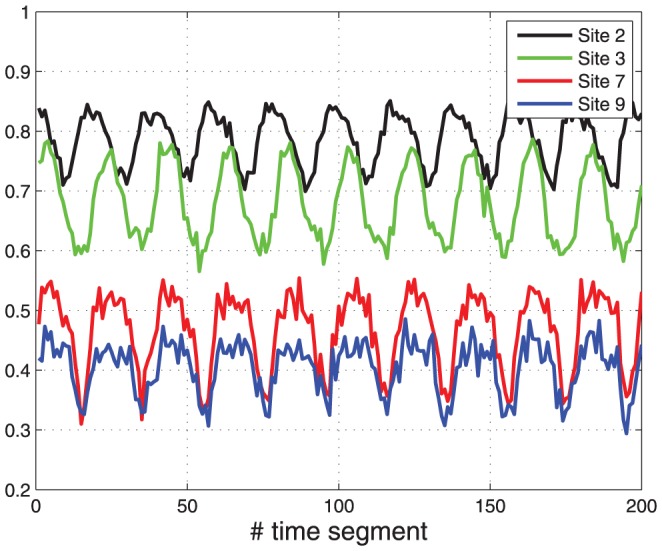
Simulation of TASEP with periodically time-varying rates. The plot shows the averaged occupancy over time segments as a function of the time segment.

Fig. 5 depicts the results. The time range 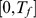 is divided into segments of length 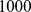 (so that there are 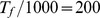 segments), and the 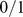 occupancy at each time step is averaged on each segment. For example, the value depicted at time segment 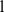 is the averaged occupancy in the time interval 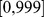. The averaged occupancy on each segment is shown in Fig. 5 for sites 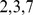, and 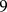. It may be seen that all the averaged occupancies entrain to the periodic excitation. In particular, they are periodic (up to the noise induced by the stochastic process) with a period of 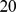 segments, corresponding to a time period of 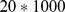 which is equal to 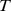 in (5).

Our simulations do suggest that some form of entrainment also takes place in TASEP. Of course, one must first rigorously define what entrainment means in a stochastic model such as TASEP.

## Discussion

Many biological and physiological processes are periodic (see, e.g., [97]), indicating periodicity in their corresponding gene expressions. For example, the cell-cycle is a periodic series of events that allows cells to replicate.

Two recent studies suggest that the protein levels of cell-cycle related genes are regulated by periodically varying tRNA levels [12], [13]. In other words, the translation-elongation mechanism *entrains* to these periodic oscillations.

To examine the plausibility of this idea, one must first consider the time constants involved; specifically, the mRNA life time and translation time should be longer than the cell-cycle period. For concreteness, consider the case of *S. cerevisiae*. The cell-cycle period in *S. cerevisiae* is less than 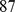 minutes [98] (cell-cycle period can be much shorter in eukaryotes; for example, it was reported that the duration of cell-cycle in early embryo of the fruit fly *D. melanogaster* is only 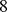 minutes [99]). In *S. cerevisiae* there are hundreds of genes with mRNA half-life larger than 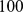 minutes (see, for example, [100]). The translation rate in *S. cerevisiae* was estimated to be higher than 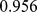 codons per second (the slowest codon is CUU) [101] with average rate over all codons of 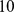 codons per second (in mouse the average codon translation rate was estimated to be around 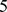 codons per second [86]). In practice, this rate can be much slower due to strong folding of the mRNA and interaction of the translated amino-acids with the exit channel of the ribosome [27]. In *S. cerevisiae* the ORF length range is between 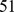 and 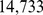 nucleotides; for example, the longest gene in yeast is MDN1/YLR106C which includes 4,911 amino acides (see http://www.yeastgenome.org). This is a huge dynein-related AAA-type ATPase (midasin) which forms extended pre-60S particle with the Rix1 complex (Rix1p-Ipi1p-Ipi3p) and acts in removal of ribosomal biogenesis factors at successive steps of pre-60S assembly and export from nucleus. This gene corresponds to an *upper* bound on the translation time of a gene that is larger than 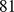 minutes (assuming a lower bound on translation rate of 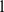 codons per second; which may be lower in practice); an estimated translated time of this protein based on mean codon translation time is 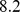 minutes. In mammals the mean codon decoding time is 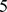 codons per second and the longest human protein (Titin – TTN) which has 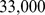 amino acids, corresponding to estimated translation time of 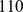 minutes. This suggests that periodically varying tRNA levels may indeed induce periodic expression levels of cell-cycle related proteins. In addition, assuming that time to steady sate is related to the translation time (at least one ribosome should finish the translation), an estimated *lower* bound on the oscillations is 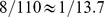 the time to reach steady state.

To rigorously analyze entrainment at the translation level, we considered the RFM under the assumption of periodic initiation rate 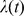 and/or periodic transition rates 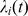 with a common minimal period 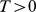. Our main result is that all the ribosome densities converge to a unique periodic solution with period 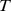. This implies in particular that the protein translation rate converges to a unique periodic function. The PRFM is thus the first computational tool providing an explanation of how periodicity can be passed from the translation to the protein level via the codon usage bias, i.e., the differences in the frequency of occurrence of different codons in the coding sequence. Specifically, according to the PRFM the distribution of codons in different open reading frames (ORFs) can affect their oscillations. For example, if the levels of certain tRNA species oscillate this will affect only genes with codons that are recognized by these tRNAs.

These results support the conjecture that oscillations of the tRNA levels and/or initiation factors, with a common period 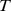, induce periodicity in the protein levels of cell-cycle genes. The assumption of variations with a common period may seem unjustified, but analysis of the PRFM shows that it is enough to oscillate a *single* tRNA level, or just the initiation rate to obtain an oscillatory behavior, with the same period, in protein synthesis. Furthermore, genes that are part of a certain pathway and/or function usually have common regulators (see, for example, [102]), and this may lead to periodic oscillations with a common period. It is important to emphasize that we do not claim here that the oscillations amplitude is necessarily “large”. There may be cases where the amplitude of the oscillations in some 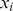 or 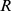 may be small and in this case the signal may “look” constant; for example, in the case that the oscillations are not in the bottleneck (in terms of translation rate) of the gene they may have a smaller effect on the translation rate.

Oscillations and entrainment also play an important role in synthetic biology [1], [8], [68]–[71]. Indeed, a major challenge in this field is scaling up to larger and more complex biological systems. One possible approach is to design networks based on an interconnection of several biological elements (modules) that synchronize to a single central clock. Entrainment is needed to achieve this. In this context, the period 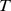 of the oscillator may perhaps be controlled and, in particular, made much shorter than the cell-cycle period. The analytic results on the PRFM may thus lead to new synthetic devices that produce periodically-varying protein levels based on oscillations in tRNA levels and/or initiation factors. Indeed, the analysis of the PRFM suggests that there are many different possible ways for generating such a periodic dynamics.

It is important to remember that oscillations in various factors, and not only the concentrations of tRNA molecules, can affect the periodic dynamics of the translation process. Among others, the oscillations in the concentrations of Aminoacyl tRNA synthetase, ribosomes (via changes in concentrations of ribosomal RNA genes and/or ribosomal proteins), elongation and initiation factors, mRNAa levels, and free amino acids may trigger an oscillatory behavior of the translation process.

One experimental approach for validating our theoretical results, and for further research of oscillations in translation is by in-vitro single-molecule fluorescence experiments (see, for example [103]–[107]). Such an experiment should encompass the different components of the translation machinery (tRNA molecules, ribosomes, elongation and initiation factors). Specifically, it will enable monitoring oscillations at the level of single ribosomal movements.

There are other types of large scale in-vitro experiments (see for example, [108]–[111]) that may be relevant. Here, crude extracts containing all the macromolecular components (70S or 80S ribosomes, tRNAs, aminoacyl-tRNA synthetases, initiation, elongation and termination factors, etc.) required for translation of RNA are prepared. To ensure efficient translation, each an extract must be supplemented with amino acids, energy sources (ATP, GTP), energy regenerating systems (creatine phosphate and creatine phosphokinase for eukaryotic systems, and phosphoenol pyruvate and pyruvate kinase for the E. coli lysate), and other co-factors (Mg2+, K+, etc.).

In-vitro experiments should enable to produce oscillations in different molecules related to the translation process (for example, the tRNA levels; similarly to the mathematical example described above) and measuring the effect of these oscillations on the ribosomal translation pattern.

The mathematical analysis performed here leads to several computational questions that deserve further study. First, our results provide little information on the periodic solution 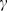. In particular, important questions are how does the amplitude and other properties of 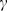 depend on the initiation and translation rates, and what is the convergence rate of the solutions to 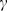. The analysis suggests that there are several possible ways to induce periodicity in the translation rate (e.g., via oscillating tRNA molecules, mRNA molecules, initiation factors, elongation factors, ribosomal RNA, etc), and it would be interesting to analyze how the periodic solution 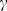 is affected by the different possible ways of inducing periodicity.

Finally, and more generally, TASEP and its variants have been used to model and analyze a large number of biological and artificial systems including biomolecular motors [112], [113], the collective motion of ants [114], traffic flow [115]–[117], ad hoc communication networks [118], and surface growth [119]. Many of these systems may be affected by periodic signals. For example, traffic flow is often controlled by periodically-varying traffic lights. It may be of interest to model and study such systems using the PRFM.

## Methods

In order to prove our main results, we first detail several known results that will be used later on.

### Preliminaries

Consider the system
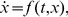
(6)evolving on a convex set 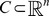. Let 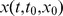 denote the solution of (6) at time 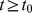 with 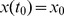 (for the sake of simplicity, we assume from here on that 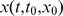 exists and is unique for all 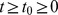).

Recall that (6) is said to be *contracting* [72] on 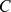 with respect to a norm 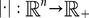 if there exists 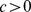 such that
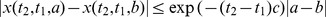
(7)for all 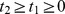 and all 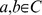. In other words, trajectories contract to one another at an exponential rate.

A standard approach for proving contraction is based on analyzing the Jacobian matrix 
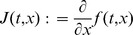
. Recall that a vector norm 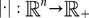 induces a *matrix norm* 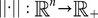 defined by
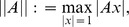
and a *matrix measure* 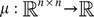 defined by
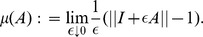
The next result, known as *Coppel*'*s inequality* (see, e.g. [120], [121]), provides a bound on the solution of a linear time-varying system in terms of the induced matrix measure.

**Theorem 3** *Consider the differential equation*

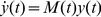
(8)


*where* 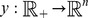, *and the matrix* 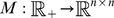 *is defined and continuous for all* 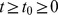. *Suppose that* 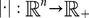 *is a vector norm and let* 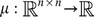 *denote the induced matrix measure. Then every solution of* (8) *satisfies*

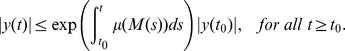



We now give an informal explanation of how this can be used to prove contraction. Suppose that there exists 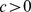 such that
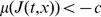
(9)for all 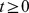 and all 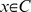, where 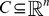 is a convex set. The distance 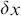 between two trajectories of (6) emanating from infinitesimally close initial conditions satisfies 
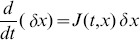
, so combining (9) and Coppel's inequality suggests that (7) holds for all 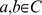. Furthermore, it can be shown that contraction implies entrainment to a periodic excitation, see e.g. [3], [72] for rigorous statements and proofs.

Consider applying these ideas to the PRFM. We can write the PRFM as 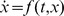, where 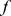 is 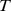-periodic, i.e. 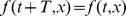 for all 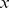 and 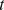. A calculation shows that the Jacobian of 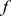 is
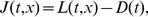
(10)where 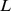 is shown below and 

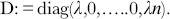


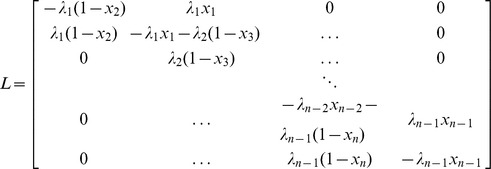



–—————————————————————————

Recall that a matrix is said to be a *Metzler matrix* if all its off-diagonal entries are non-negative. Note that 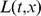 is Metzler, tridiagonal, and with zero sum columns for all 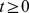 and all 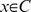.

It is well-known ([122], Chapter 3) that the induced matrix measure corresponding to the 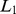 vector norm is
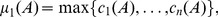
(11)where
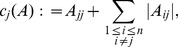
(12)
*i.e.*, the sum of the entries in column 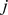, with non diagonal elements replaced by their absolute values. Of course, if 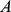 is Metzler then one can take 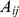 instead of 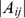 in (12).

Calculating 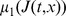 for the PRFM shows that when 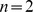,
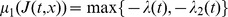


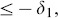
so in this case we have contraction with respect to the 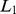 norm. However, for 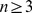, 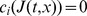 for 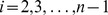, and 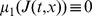. Intuitively, this means that the PRFM is on the “verge” of contraction with respect to the 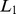 norm, but this is not enough to prove entrainment. Furthermore, when 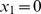 and 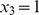 all the entries in the second column of 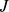 are zero, and this implies that the PRFM is *not* a contraction on 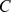 with respect to *any* norm (as a necessary condition for contraction is that 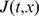 is a Hurwitz matrix for all 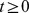 and all 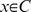).

### Proof of Main Result

For two vectors 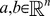, we write 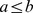 if 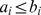 for 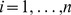. Let 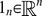 denote the vector with all entries equal to one. For 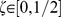, define
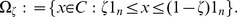
Note that 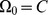, and that 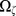 is a strict subcube of 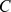 for all 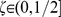. The next result shows that the trajectories of the RFM always enter such a strict subcube, and then remain in it.

**Proposition 1** *Consider the PRFM. Fix arbitrary* 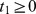, 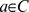, *and* 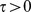. *There exists* 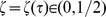, *with* 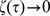 *as* 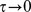, *such that*

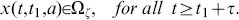
(13)


*Proof*. See the section Additional Proofs.

Recall that the PRFM is not a contraction on 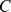 with respect to any norm. The next result shows that contraction does hold on any strict subcube of 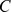. The proof, given in the Additional Proofs section, uses a suitable diagonal scaling of the 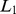 norm.

**Proposition 2** *Fix an arbitrary* 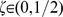. *Then the PRFM is contracting on* 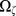.

Note that all the proofs up to this point (given in the section Additional Proofs) do not rely on the assumption that the rates are periodic functions. We can now prove our main result.

*Proof of Theorem 2*. Recall that the excitation is periodic with period 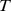. Let 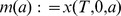, *i.e.*, 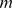 maps the initial condition 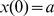 to the solution of the PRFM at time 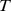. Then 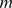 is continuous and maps 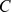 to 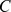, so by the Brouwer fixed point theorem (see, e.g. [123]) there exists 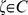 such that 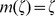, i.e. 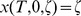. This implies that the PRFM admits a periodic solution 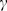 with period 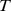. It follows from Proposition 1 that 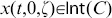 for all 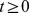. We already know that every trajectory enters some strict subcube of 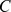, that this subcube is an invariant set, and that in this subcube contraction holds. Thus, every trajectory converges to the periodic solution emanating from 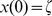. In particular, there cannot be two distinct periodic solutions. This completes the proof of Theorem 2. 

Note that the reasoning above does not rule out the possibility that
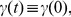
(14)for all 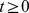, i.e. that the periodic trajectory is just a fixed point. To study this case, assume that a fixed point 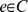 indeed exists. Then (1) yields
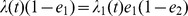


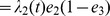


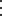


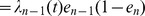


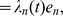
for all 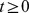. Thus, (14) is possible only in the rather special case where all the rates are equal, up to multiplication by a non-negative scalar.

### Additional Proofs

This section includes the proofs of several results stated above. We begin by stating and proving two auxiliary results. The next subsection describes a result that will be used to prove Proposition 1.

### Repelling Boundaries and Persistence

**Lemma 1** *Consider a time-varying system*

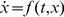
(15)


*evolving on a subset of* 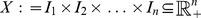, *where each* 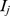 *is an interval of the form* 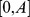, 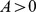, *or* 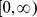. *Suppose that the time-dependent vector field* 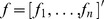 *has the following boundary-repelling property*:

**(BR)** *For each* 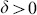 *and each sufficiently small* 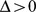, *there exists* 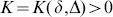 *such that, for each* 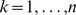 *and each* 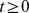, *the condition*

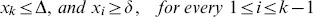
(16)(for 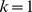, *the condition is simply* 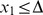)

*implies that*

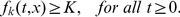
(17)


*Then given any* 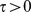 *there exists* 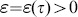, *with* 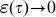 *as* 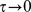, *such that, for every solution* 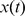, 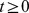, *it holds that*

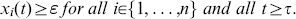



In other words, the conclusion is that after an arbitrarily short time every 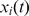 is separated away from zero.

*Proof of Lemma 1*. Pick any 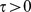. Let 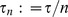. We proceed by induction: for each 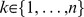 we will define an 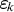 and show that for every solution 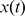, 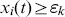 for every 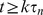 and every 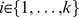. Then 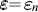 gives the result. Pick any fixed 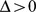 small enough that **(BR)** holds, and let 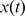 be any given solution. From here on, we write just 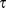 instead of 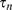.

Consider first the case 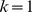. Let 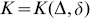 (for any arbitrary 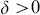) and define 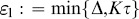. Let 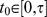 be such that 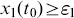. Such a 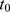 exists, since by property **(BR)**, 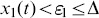 for all 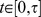 would imply that 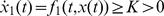 for all 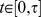, which in turn implies 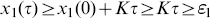, contradicting 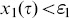. We claim that also 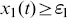 for every 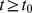. Indeed, suppose otherwise. Then, there is some 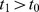 such that 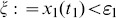. Let
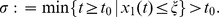
As 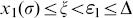, property **(BR)** says that 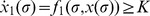, so it follows that 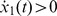 on an interval 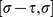, for some 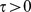. But then 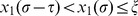, which contradicts the minimality of 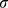. Thus 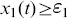 for all 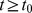, and in particular for all 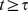.

Now by induction, consider 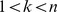, and suppose that 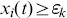 for all 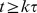, and every 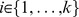. We must define 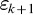 so that 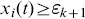 for all 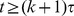 and every 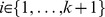. Let 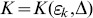 in **(BR)**, and define 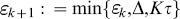.

Let 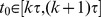 be such that 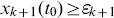. Such a 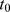 exists, since by property **(BR)**, (using that 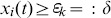 for all 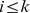 and 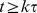) 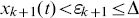 for all 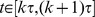 would imply that 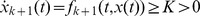 for all 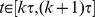, which in turn implies 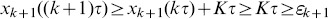, contradicting 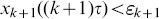.

We claim that also 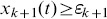 for every 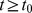. Indeed, suppose otherwise. Then, there is some 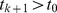 such that 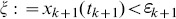. Let
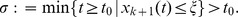
As 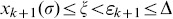, and also 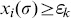 (because 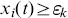 for all 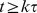, and 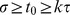), we may apply property **(BR)**, which says that 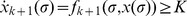, so it follows that 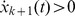 on an interval 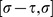, for some 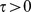. But then 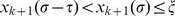, which contradicts the minimality of 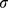. Thus 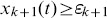 for all 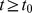, and in particular for all 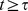. By the definition of 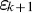 and the induction hypothesis, we also have 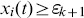 for all 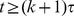 and every 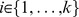. 

We can now prove Proposition 1. We begin by showing that the PRFM satisfies property (BR) in Lemma 1 on 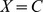. Fix an arbitrary 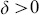. Consider the case 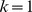. If 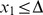 then
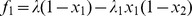


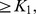
where 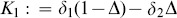. Now pick 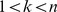. If 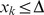 and 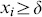 for 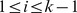 then
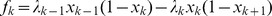


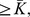
where
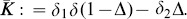
Finally, if 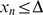 and 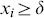 for 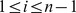 then
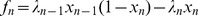


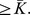
Thus, (17) holds for 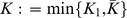 and clearly 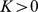 for all 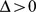 sufficiency small. Thus, the PRFM satisfies **(BR)**. Pick an arbitrary 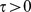. Applying Lemma 1 implies that there exists 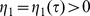 such that
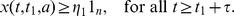
Define 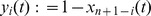, 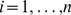. It is straightforward to verify that the dynamics of the 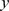-system is just that of the PRFM, up to a reordering of the rates 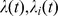. This implies that the 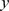-system also satisfies property **(BR)**, so there exists 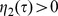 such that
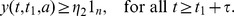
Thus, (13) holds for 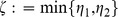. This completes the proof of Proposition 1. 

**Remark 2** Note that the proof above shows that the requirement that the rates are uniformly separated from zero by 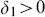 (see (4)) cannot be omitted. Indeed, if 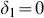 then 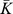 is no longer positive. In fact if we allow the rates to vanish identically then Proposition 1 does not hold. For example, suppose that 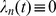. Then 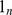 becomes an equilibrium point of the RFM, and so (13) does not hold for 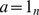.

The next subsection includes a result on diagonal scaling of a tridiagonal matrix. This will be used to prove that the PRFM is a contraction on 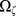, 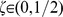.

### Diagonal Scaling of a Tridiagonal Matrix

Let 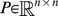 be an invertible matrix. Define a vector norm 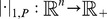 by 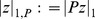. The induced matrix measure is 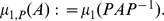


**Theorem 4** *Suppose that* 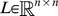 *is a tridiagonal matrix with zero sum columns, and that there exist* 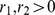 *such that*

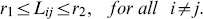
(18)
*Then for each* 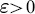 *there exist*

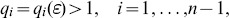
(19)
*such that for the matrix*

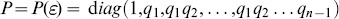

*and the matrix measure*

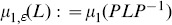

*the following properties hold*:

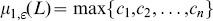
 where
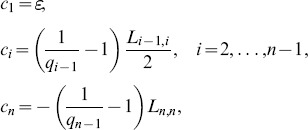
(20)
*and there exists* 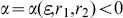 *such that* 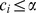 *for* 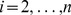.
2. *if* 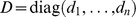 *is a non-negative diagonal matrix, with* 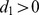, *then*

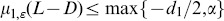


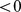

*for all* 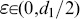.

*Proof of Theorem 4*. Since 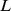 is tridiagonal with zero column sums, we can write
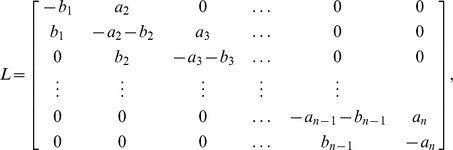
with
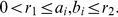
(21)Therefore
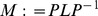


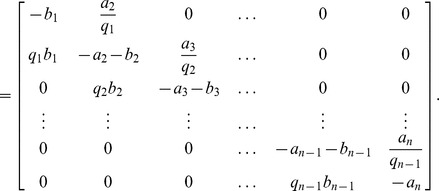
Let 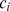 denote the sum of the elements in column 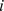 of 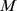, with off diagonal elements taken with absolute value. Since the off diagonal elements of 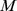 are non-negative,
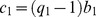


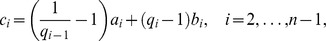


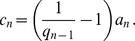
Pick any 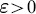 and define, recursively:
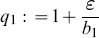


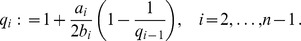
Then clearly (20) holds. Using (21) yields 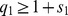, where 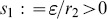. Combining this with the definition of 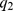 and (21) implies that there exists 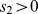 such that 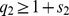. Proceeding in this fashion yields an 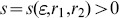 such that 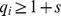 for all 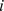. By (20), this implies that there exists 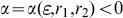 such that 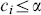 for 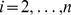. This completes the proof of Theorem 4. 

We can now prove Proposition 2. Pick 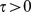, 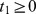, and 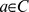. By Proposition 1, there exists 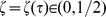 such that (13) holds. This implies that the off diagonal elements of 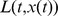 satisfy
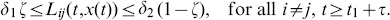
Recall that the Jacobian of the PRFM is 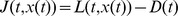, with 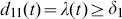. Let 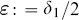. By Theorem 4, there exists a matrix 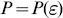, and a scalar 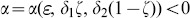, such that 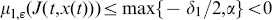 for all 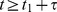. Thus, the PRFM is contracting on 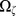 with respect to the norm 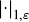. This completes the proof of Proposition 2. 
