# Correction: Entrainment to Periodic Initiation and Transition Rates in a Computational Model for Gene Translation

**DOI:** 10.1371/journal.pone.0101909

**Published:** 2014-06-27

**Authors:** The PLOS ONE Staff

The authors would like to include additional funding information. The Funding statement should read:

EDS's work is supported in part by grants NIH 1R01GM086881 and 1R01GM100473, and AFOSR FA9550-11-1-0247. The research of MM is partly supported by the ISF. All other sources of support are internal (Tel-Aviv University and Rutgers University). The funders had no role in study design, data collection and analysis, decision to publish, or preparation of the manuscript.
